# Exposure to respirable dust among workers fabricating aluminium trihydroxide-containing synthetic countertops

**DOI:** 10.1038/s41598-021-00814-5

**Published:** 2021-10-27

**Authors:** Denis Vinnikov, Paul D. Blanc, Aizhan Raushanova, Arailym Beisbekova, Jerrold L. Abraham, Yelena Zlobina

**Affiliations:** 1 Al-Farabi Kazakh National University, 71 al-Farabi avenue, 050040 Almaty, Kazakhstan; 2 Peoples’ Friendship, University of Russia, RUDN University), Moscow, Russian Federation; 3 University of California San Francisco, San Francisco, CA USA; 4 Asfendiyarov Kazakh National Medical University, Almaty, Kazakhstan; 5 SUNY Upstate School of Medicine, Syracuse, NY USA

**Keywords:** Epidemiology, Risk factors

## Abstract

The aim of this study is to characterize personal exposure of workers to respirable particulate matter (PM) generated in cutting and other fabrication activities when fabricating acryl polymer/aluminium trihydroxide synthetic countertops. We collected 29 personal full-day samples of respirable PM from three workers in a small private workshop. We tested differences between- and within-worker variances of mass concentrations using the Kruskall-Wallis test. We used segmented regression to test the means and medians 15-min interval concentrations changes over time and to identify a breakpoint. Respirable PM concentrations ranged nearly 100-fold, from 0.280 to 25.4 mg/m^3^ with a median of 2.0 mg/m^3^ (1-min concentrations from 13,920 data points). There were no statistical difference in daily median or geometric mean concentrations among workers, whereas the concentrations were significantly higher on days with three versus two workers present. The 15-min median concentrations (n = 974 measures) increased until 2.35 h (beta 0.177; *p* < 0.05), representing a 0.70 mg increase in exposure per hour. This was followed by a plateau in concentrations. The high levels of respirable PM we observed among workers fabricating aluminium trihydroxide-containing synthetic countertops highlight an unmet early prevention need.

## Introduction

There is widening recognition that engineered stone countertops present industrial hygiene challenges to successfully control potential health hazards, primarily due to their silica content^1–4^. Hygiene and health concerns linked to other, non-silica synthetic countertop materials have received less attention, despite their widespread use. In particular, aluminium trihydroxide (ATH)-containing synthetics comprise an important type of material to be considered in the exposure assessment of countertop fabrication. For example, chronic inhalational exposure to dust from cutting and sanding Corian™, a major brand of aluminium hydroxide/trioxide ATH containing synthetic countertop, has been implicated in at least one well-documented case report of lung fibrosis^5^. Experimental animal data further support the potential of such inhalation to be a respiratory hazard^6^.

Exposure simulations indicate that working with ATH-containing synthetic countertops can lead to substantial airborne dust. In one set of simulated exposures, dust generated from cutting Corian™ was 32% in the respirable size range, contained 82% aluminium hydroxide, and had a respirable mass concentration as high as 1.52 mg/m^3^^3, 7^. A subsequent report using a similar simulated approach found that nearly twice as much of the dust produced (59%) was in the respirable range^8^. Very little is known, however, about real-world exposure patterns among workers fabricating such materials, including day-to-day and across-shift variability in working conditions and resulting exposures. The aim of this study was to characterize personal exposure of workers to respirable particulate matter generated in cutting and other fabrication activities when working with acryl polymer/aluminium oxide (off-patent Corian) in a typical, small countertop operation.

## Results

**Figure 1:**
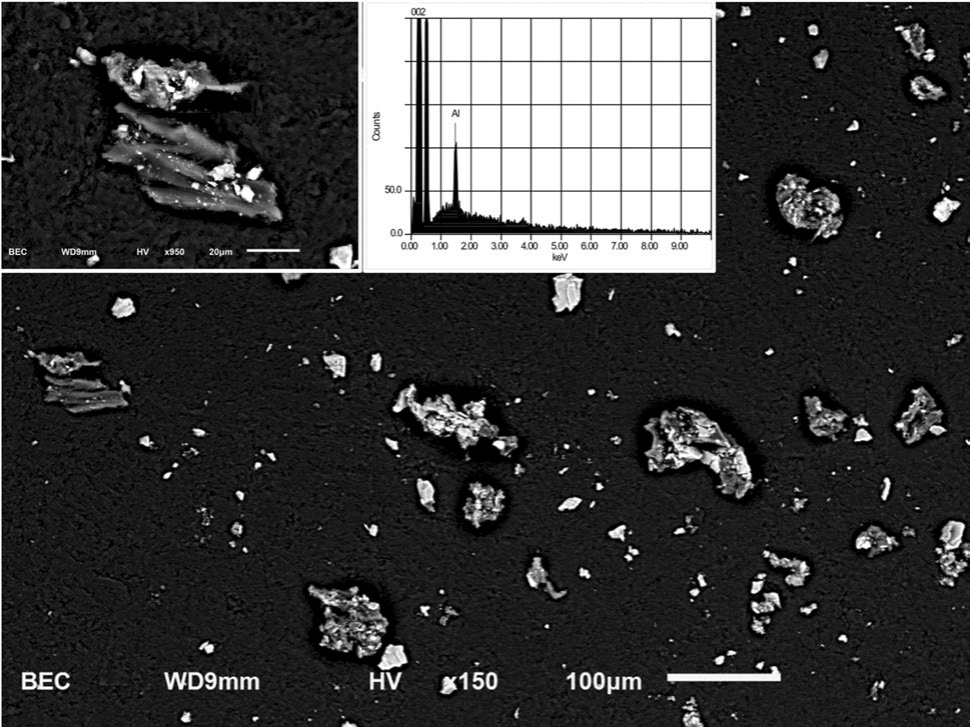
Scanning electron microscope images and energy dispersive x-ray spectrum of counter dust collected by area sampling. Brightness in these backscattered electron images is dependent on atomic number. Particles are of a range of sizes and shapes consistent with grinding/sanding operations and show a mix of bright smaller particles and larger less bright fragments. Left inset shows detailed morphology of selected larger fragments. Right inset shows a spectrum typical of the brighter particles, confirming aluminium as the major detectable element. No elements other than carbon and oxygen are detectable in the less bright phase particles. Magnification scale markers indicate 100 µm and 20 µm.

Atomic absorption analysis of dust showed that 40.4% was aluminium by elemental analysis, corresponding based on molecular weight to 76.1% aluminium oxide. Other elemental components detected were all in substantially lower concentrations: 0.53% calcium, 0.15% magnesium, 0.14% iron, 0.10% sodium, 0.05% barium, 0.03% potassium and 0.01% strontium. Trace Zn (0.005%) and Cu (0.001%) were also detected. Images from the scanning electron microscopy (SEM) with energy dispersive x-ray spectroscopy (EDS) analysis are shown in Fig. 1. The predominant elemental finding was aluminium (not distinguishable in this method between aluminium oxide vs. trihydroxide). Trace silica was present in only one of multiple particles analyzed. Other amorphic particles were consistent with an organic polymer substance that could not otherwise be characterized.

Workday duration in workshop fabrication ranged from 309 min (5.1 h) to 649 min (10.8 h) (median 494 (interquartile range (IQR) 30) minutes). There were no statistical differences among the three workers in the workday duration (Kruskall–Wallis *p* = 0.30). The mean of all 1-min respirable PM concentrations based on 13,920 data points was 2.66 ± 2.31 mg/m^3^; the median was 2.0 mg/m^3^. Recorded respirable one-minute PM concentrations ranged nearly 100-fold: from 0.280 to 25.4 mg/m^3^. The daily total exposures of each worker varied by nearly an order of magnitude. For worker 1, for example, the daily median ranged from 0.751 to 7.185 mg/m^3^.

Table 1 shows the observed minimum, maximum, and central tendencies, and 75^th^ percentile for each of 29 daily samples among the three workers. For worker 1, on four days 25% of the samples were above 5 mg/m^3^; for worker 2, there was only one such day in which 25% of samples were above 5 mg/m^3^; and for worker 3 there were no days with 25% of samples above 5 mg/m^3^. Altogether, peak sampling concentrations exceeded 10 mg/m^3^ on 11 of 29 days.

**Table 1:** Respirable particulate matter mass concentrations from daily personal samples in three workers in mg/m^3^.

	Min	Max	Mean	Median	75^th^ %tile	Geometric mean
Worker 1, All Days	0.280	25.400	3.351	2.030	4.650	2.205
Day 1	0.376	12.600	1.919	1.650	2.555	1.567
Day 2	0.280	18.800	4.281	4.360	5.940	3.151
Day 3	0.362	9.080	1.057	0.961	1.110	0.943
Day 4	0.497	7.140	2.473	2.370	3.235	2.097
Day 5	0.465	5.820	1.350	0.997	1.690	1.224
Day 6	0.339	5.110	0.811	0.751	1.080	0.740
Day 7	0.299	6.630	1.940	1.690	2.440	1.708
Day 8	0.491	14.300	4.940	4.730	6.140	4.315
Day 9	2.160	25.400	7.932	7.185	9.910	6.960
Day 10	2.440	19.400	6.810	6.215	8.438	5.998
Worker 2, All Days	0.505	15.200	2.278	1.560	3.010	1.768
Day 1	1.600	8.070	3.598	3.220	4.475	3.395
Day 2	2.170	12.900	4.768	4.095	5.685	4.395
Day 3	0.800	10.500	1.281	1.190	1.410	1.216
Day 4	0.978	6.840	2.947	2.880	3.220	2.710
Day 5	1.020	5.690	1.725	1.310	2.010	1.555
Day 6	0.511	1.850	0.859	0.708	1.088	0.798
Day 7	0.505	3.660	0.706	0.637	0.733	0.678
Day 8	0.586	2.540	1.432	1.510	1.690	1.372
Day 9	0.803	15.200	3.792	3.175	4.240	3.209
Day 10	1.230	8.530	1.676	1.610	1.780	1.641
Worker 3, All Days	0.579	24.600	2.336	2.090	2.640	2.114
Day 1	1.750	20.700	3.616	2.560	4.228	3.133
Day 2	1.080	5.550	2.600	2.560	3.015	2.471
Day 3	0.579	4.660	1.931	1.740	2.218	1.868
Day 4	1.150	9.210	2.314	2.250	2.780	2.158
Day 5	0.897	3.880	1.888	1.850	2.228	1.803
Day 6	1.320	15.800	2.866	2.090	3.350	2.564
Day 7	0.907	3.770	2.075	2.090	2.540	1.950
Day 8	0.900	24.600	1.622	1.360	1.710	1.458
Day 9	1.080	3.380	2.107	2.175	2.380	2.058

**Figure 2:**
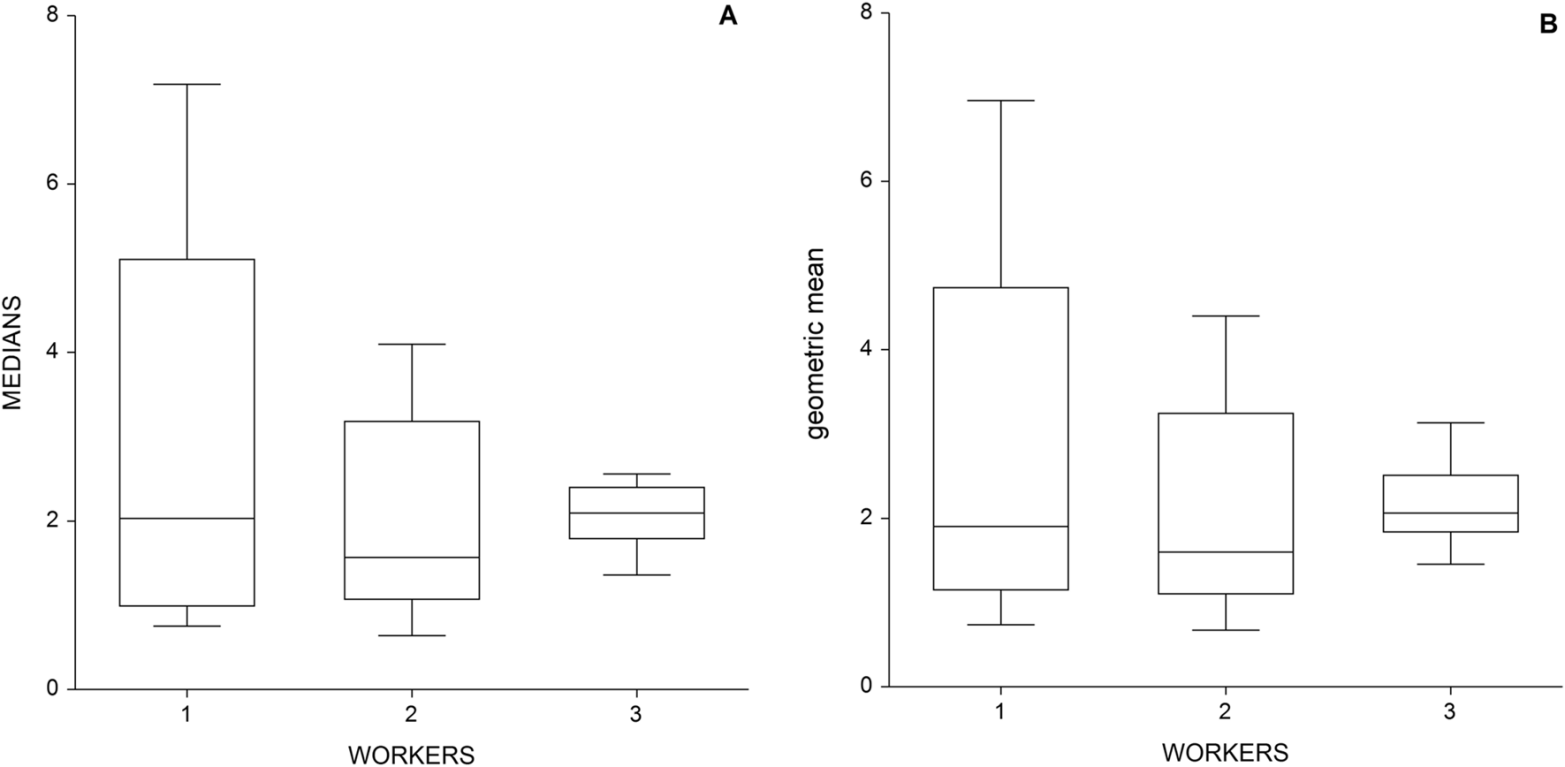
The median (**A**) and geometric mean (**B**) concentrations of respirable PM in three included workers.

Figure 2 illustrates the pooled data by worker. For Panel A, there was no statistically significant difference among the three workers in daily median concentrations (Kruskall–Wallis *p* = 0.62) or, as shown in Panel B, in geometric means (Kruskall–Wallis *p* = 0.73).

**Figure 3:**
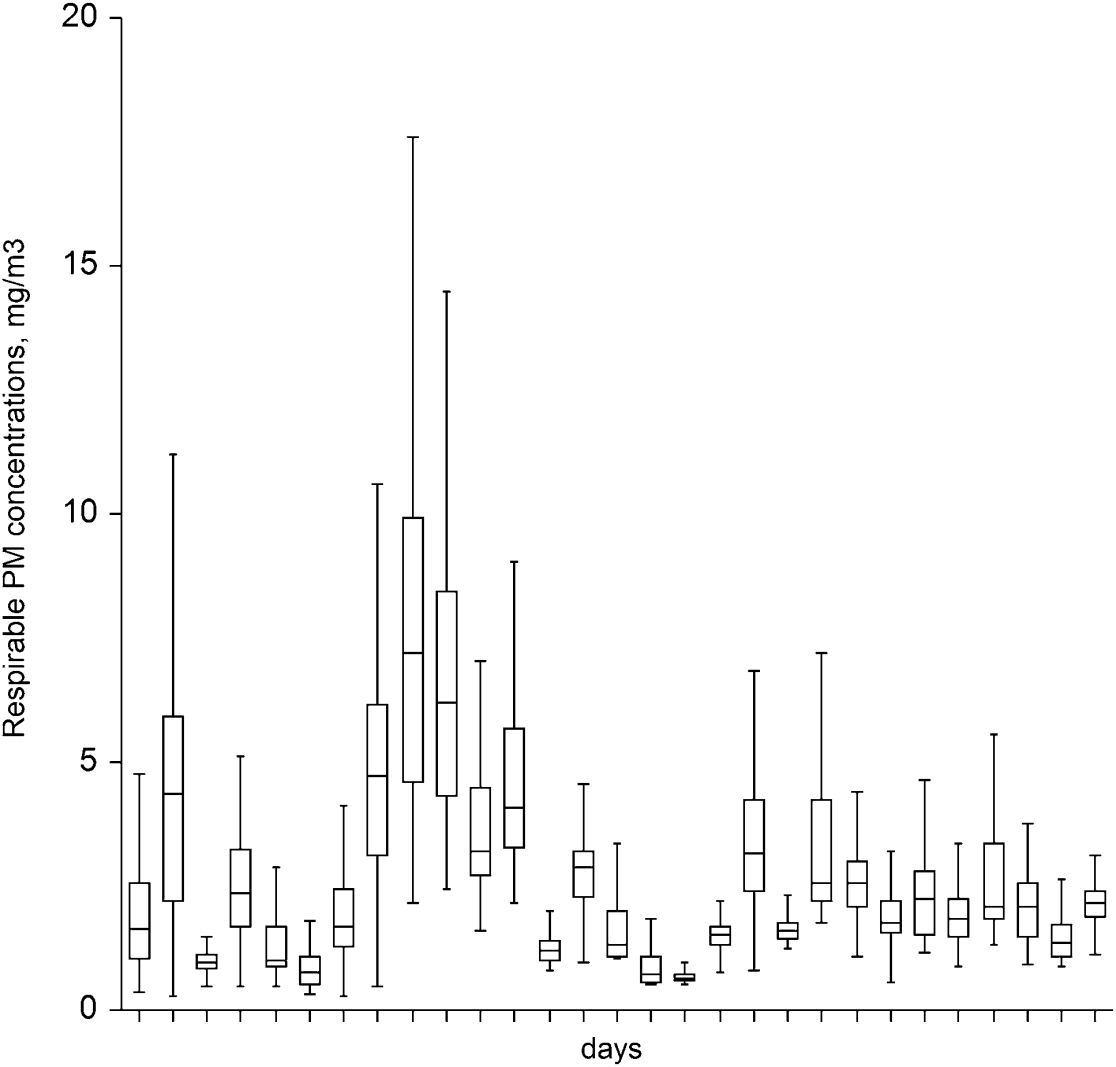
Medians with the corresponding IQR as boxes and 1.5*IQR as whiskers plotted for each working day.

We tested whether measured concentrations differed by the number of workers present. As shown in Fig. 3, the concentrations were significantly higher on the days with a greater number of workers present (Kruskall–Wallis *p* < 0.001). When one worker was absent compared to days with full staffing on site, there were significantly lower concentrations for the former (median 1.65 (IQR 1.41) mg/m^3^ vs. 2.21 (IQR 2) mg/m^3^; *p* < 0.01) The corresponding geometric mean concentrations were 1.77 and 2.20 and mg/m^3^, respectively.

**Figure 4:**
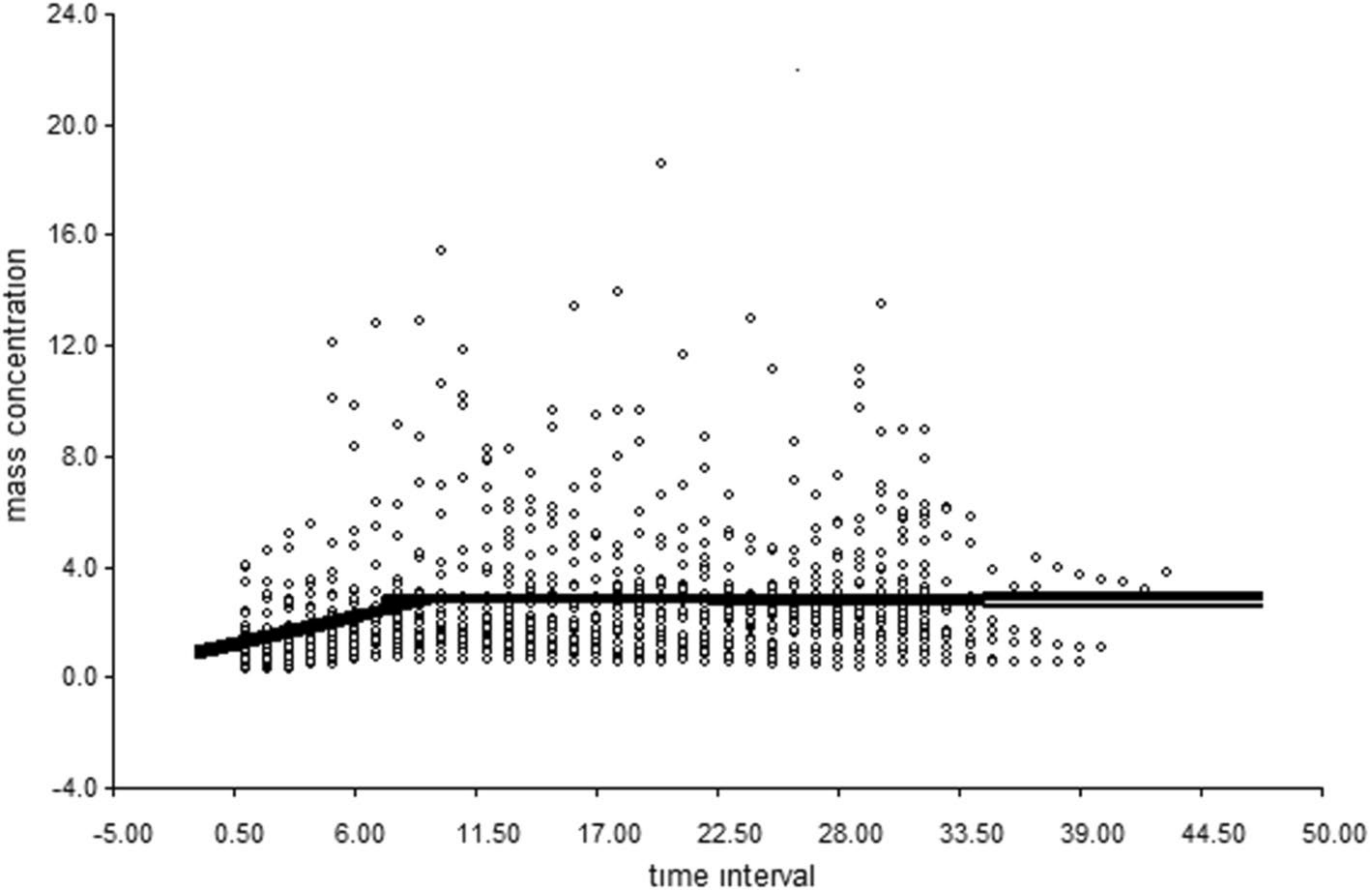
Respirable PM concentrations trend throughout the workday. There is a growing trend until the breakpoint with a subsequent straight line.

The segmented regression analysis of 15-min medians from all 29 samples (n = 974 measures) identified two distinct predictive lines: the first with increasing mass concentration until a breakpoint at 9.4 15-min intervals (2.35 h), followed by a flat trajectory plateauing thereafter (Fig. 4). The beta coefficient of the regression up to 2.35 h was 0.177 (*p* < 0.05) equating to a 0.70 mg increase in exposure per hour or a total of 1.65 mg increase over the initial 2.35-h time period. Re-estimating the segmented regression including a variable for the presence of a full versus partial workforce did not substantively change the estimated breakpoint or the slope of the pre-breakpoint line.

## Discussion

Our findings document that personal breathing zone respirable dust exposure in acryl polymer/aluminium ATH oxide synthetic countertop fabrication can be substantial. Based on 29 full working-day samples for three workers under routine working conditions, respirable PM concentrations ranged nearly 100-fold, from 0.280 to 25.4 mg/m^3^. The majority of the workdays reached peak exposures over 10 mg/m^3^. Exposure increased over the initial working hours and then plateaued, yielding median values of 2.0 mg per cubic meter for two of three workers sampled. The peak exposures were considerably greater than the 75th observed percentile values while the median concentrations among the three workers, each sampled on separate days, were similar (2.0, 1.6, and 2.1 mg/m^3^). The pattern of variability within but not across workers is consistent with periods during the working day performing heavy dust-generating tasks.

Timely identification of high levels of personal exposure to aluminium or ATH-containing and acryl polymer respirable dust is important because it may potentially lead to a progressive pulmonary fibrosis, as described in a case report of a 64 year-old man who worked cutting synthetic solid-surface material, identified as Corian^5^. The presence of excess aluminium (as both ATH and aluminium oxide) was verified in that report by tissue analyses. An earlier report of aluminium oxide-exposed workers from a plant engaged in the production of Al_2_O_3_ abrasives from alundum ore, documented pulmonary fibrosis without tissue evidence of asbestosis or silicosis, but with aluminium oxide and alloys present in high tissue concentrations^9^.

The fabrication of artificial countertops has garnered new scrutiny due to inherent silica hazards. Although occupational lung disease due to aluminium-ATH containing synthetics is not nearly as well established, the risk is not purely theoretical. Experimental animal data support the potential toxicity of such materials. Moreover, simulated exposure data have shown that dust generation for fabricating Corian can be substantial^7, 8^.

It is in that context that our findings should be considered. In a real-world setting of routine operation, we demonstrated substantial exposures to aluminium-ATH-containing dusts on a nearly daily basis. Our-real world aerosol concentrations yielded data that differed somewhat from observations reported from simulated exercises. Differences may be attributable to multiple factors not captured in simulations, such as the size and configuration of the workshop, the number of workers present, and the pace of work, to name a few salient variables. The operation we studied was relatively small, but is likely to be fairly typical of countertop installation work in many places. Moreover, because exposures were higher when all three workers were present, we may actually have underestimated the exposure potential. We found higher levels of exposure on days when more workers were present, indicative of more aerosol produced within a fixed space.

We limited our study of personal breathing zone exposure to respirable range particulate by mass and did not ascertain the full-size distribution. Nonetheless, we would not anticipate that ultrafine particulates would be relevant to mechanical sawing or grinding (as opposed to welding or flame cutting), whereas the respirable range concentrations can be presumed critical in dust control in countertop fabrication. We confirmed the dominant presence of aluminium as the key metallic constituent of the dust both by atomic absorption and by SEM/EDS, although this could be either aluminium oxide or trihydroxide. Unlike the fabrication of quartz synthetics, silica exposure was minimal, as would be anticipated. One limitation in our characterization of the dust it that the atomic absorption did not report and EDS cannot detect beryllium, a respiratory hazard that can be present in very low trace amounts in aluminium-containing materials. A more important limitation is that we were not able to characterize the precise organic component of the material used, for example, methacrylate or a related acryl polymer. Other analytic approaches, such as Raman spectrography, might address this question in further research. An additional limitation of our exposure assessment is that we only measured exposure in one worker at time and thus do not have simultaneous observations to cross-compare. This may be relevant given that maximum exposure values were quite elevated. We addressed this distribution by considering the median and 75th percentile values as reliable metrics of exposure.

This study has distinct implications for policy in the occupational hygiene and occupational health fields. Because the labor market of synthetic solid-surface material is unregulated in Central Asia and likely elsewhere, more quantitative analyses are needed to fully understand the magnitude of exposure. Moreover, since the number of people employed in such workplaces and thus exposed is unknown, a clear public health action plan is indicated in order to better estimate the scale of the problem. In addition, some of those exposed may warrant medical surveillance for the early detection of respiratory impairment and disease. Yet we should not wait for potential illness to emerge. Engineering dust control measures, such as water suppression and appropriate exhaust ventilation, should be instituted. Such steps are indicated even if this was approached as simply a relatively benign “nuisance” dust, which it should not be presumed to be.

In conclusion, we have documented personal breathing zone exposure to high levels of respirable PM among workers fabricating aluminium-ATH containing synthetic countertops. Given that the labor market of workers employed for these workplaces is unregulated, identification of exposed workers is essential for early prevention of respiratory complications. Timely exposure control measures in such operations are warranted.

## Materials and methods

### Study site

The worksite that we studied is a privately-owned countertop preparation workshop located in an urban residential neighborhood in Almaty, Kazakhstan. This workplace occupies a single, one building structure attached to a former private residence. This workshop is comprised of a 400 m^2^ open space without partitions. The enterprise employs three shop floor workers and one manager. At the workshop site, employees carry out countertop fabrication comprised of cutting and sanding/polishing at the workshop site. They also work installing countertops in the field. Field work requires two to three residential visits for measurements and final installation.

At the workshop, cutting and sanding/polishing is carried out using power equipment without any water-based dust suppression. The workshop has no general or directed exhaust ventilation in place. The typical workday is eight hours, but may extend up to 12 h depending on the workload. The workshop operates five days a week. Slabs of feedstock synthetic material for fabrication is imported from China in large rectangle slabs, approximately 3.6 by 0.76 m in size, 5 cm thick. The full chemical composition of the materials, including any silica content, is not specified in the documents accompanying the products, but they all are described as being an “acryl polymer”. The brand names of the products used are: LG Hi-Macs, Samsung Staron, and Grandex. All methods were carried out in accordance with relevant guidelines and regulations, including Declaration of Helsinki. All experimental protocols were approved by the Committee on Bioethics of the Faculty of Healthcare and Medicine of al-Farabi Kazakh National University (approval #96/2020). Informed consent was obtained from all subjects in this study.

### Exposure assessment

We assessed the respirable fraction of the dust produced during cutting using a direct reading apparatus, the TSI SidePak AM510 (TSI, USA). The device uses light-scattering technology to quantify mass concentration of the dust every second. A single device was mounted to the waist belt of one of the participating workers (n = 3) on each day of observation. Air was pumped into the device through Dorr-Oliver cyclone in order to separate respirable fraction. The internal pump operated at 1.7 L per minute. The cyclone was placed in a breathing zone, fixed to the worker’s shirt collar with a clip. We recorded the 1-min averaged mass concentrations. We zeroed the device every day prior to the beginning of fabrication at workshop. We did not perform air sampling during countertop installation in the field (which always took place in the mornings).

Because workdays differed in length, there was no fixed sampling time. All three workers employed in this workshop were asked to provide ten samples each on consecutive workdays. One of 10 samples from worker #3 was not recorded properly and thus was excluded; therefore, we analyzed 29 daily samples in total, each collected for one worker on the sampling day in question. On the majority of days, all three workers were simultaneously in the workplace cutting the slabs; however, worker #3 took ten days off during the period of sample collection. At the end of each day, we downloaded recordings from the monitor being used that day to a personal computer, allowing calculation of daily means, medians, geometric means and maximal and minimal mass concentrations.

### Chemical composition analysis

We used standard atomic absorption (Agilent 7500a, Japan) to carry out elemental analysis of the particulates generated in fabrication collected in an area sample during one working day. Silica content was not quantified in this analysis. To further characterize airborne dust from this sample was also analyzed using scanning electron microscopy (SEM) with energy dispersive X-ray spectroscopy (EDS).

### Statistical analysis

We analyzed minimal, maximal, mean, median and geometric mean values of 1-min mass concentrations from each day (29 days in total). We tested normality using Shapiro–Wilk test, which indicated that these data were non-normally distributed, being characterized by left-skewedness. For this reason, we present data medians and IQR unless otherwise specified, and used non-parametric testing wherever relevant. We used the Kruskall-Wallis test to assess differences in workday duration and the cumulative daily dust sampled among the three workers and whether the variance among days exceeded within day variation. In addition, we also dichotomized between days with a full complement of three staff in the workplace versus those days with only two workers present were at site, using the Mann–Whitney U-test to assess whether there was a statistical difference in dust concentrations between them.

To assess time-dependent concentration changes over the workday for 29 sampling days, we calculated means and medians of every 15-min interval from every sampling day, yielding from 21 to 43 data points over each workday (depending on its duration). Visual inspection of a data graph revealed a non-linear association of the concentration over time, showing a steady rise followed by a plateau. To address this, we applied segmented (piecewise) regression to test whether there was a statistical breakpoint in time-dependent concentrations. This regression analysis confirmed the presence of a breakpoint and its estimated time point along with separate beta coefficients and associated 95% confidence intervals (CIs) for two resulting regression equations (prior to and after the breakpoint). These regressions, in which time interval was an independent predictor, were re-estimated including an additional independent dichotomous predictor representing whether full staff (n = 3 workers) versus a partial workforce (n = 2 workers) was present.

All analytical procedures were completed in NCSS 2020 (Utah, USA) with the exception of the segmented regression analysis, which used SegReg software (www.waterlog.info).

## Data Availability

All data generated or analysed during this study are included in this published article.
